# Treatment patterns and burden of complications associated with sickle cell disease: A US retrospective claims analysis

**DOI:** 10.1002/jha2.575

**Published:** 2022-10-06

**Authors:** Deepa Manwani, Arthur L. Burnett, Jincy Paulose, Glorian P. Yen, Tanya Burton, Amy Anderson, Sara Wang, Soyon Lee, Santosh L. Saraf

**Affiliations:** ^1^ Albert Einstein College of Medicine The Children's Hospital at Montefiore The Bronx New York USA; ^2^ Department of Urology The Johns Hopkins University School of Medicine Baltimore Maryland USA; ^3^ Novartis Pharmaceuticals Corporation East Hanover New Jersey USA; ^4^ Optum Life Sciences Eden Prairie Minnesota USA; ^5^ Sickle Cell Center Division of Hematology and Oncology University of Illinois Hospital and Health Sciences System Chicago Illinois USA

**Keywords:** healthcare costs, opioid use, organ dysfunction, retrospective studies, sickle cell disease, United States

## Abstract

Complications associated with sickle cell disease (SCD) that are highly impactful for patients but until recently have been less understood include priapism, nephropathy, and neurologic injury. We conducted a retrospective study using US administrative claims data from July 01, 2013 through March 31, 2020 to analyze incidence of these complications, SCD treatment patterns, and healthcare resource utilization (HCRU) and costs among 2524 pediatric and adult patients with SCD (mean [SD] age 43.4 [22.4] years). The most common treatments during follow‐up were short‐acting opioids (54.0% of patients), red blood cell transfusion (15.9%), and hydroxyurea (11.0%). SCD complications occurred frequently; in the overall population, the highest follow‐up incidences per 1000 person‐years were for acute kidney injury (53.1), chronic kidney disease (40.6), and stroke (39.0). Complications occurred across all age groups but increased in frequency with age; notably, acute kidney injury was 69.7 times more frequent among ages 65+ than ages 0–15 (*p* < 0.001). Follow‐up per‐patient‐per‐month HCRU also increased with age; however, all‐cause healthcare costs were similarly high for all age groups and were driven primarily by inpatient stays. Patients with SCD across the age spectrum have a high burden of complications with the use of current treatments, suggesting unmet needs for treatment management.

## 1 INTRODUCTION

Sickle cell disease (SCD) is an inherited blood disorder that results in malformation of red blood cells, leading to hemolytic anemia and vaso‐occlusion with associated pain, tissue ischemia, and acute and chronic organ damage [1, 2]. SCD occurs in about one of every 365 African‐American births and one of every 16,300 Hispanic‐American births, affecting an estimated 100,000 Americans [3]. The clinical manifestations of SCD negatively affect quality of life, disrupt daily activities, and reduce life expectancy [4, 5, 6]; it is estimated that individuals with SCD lose more than three decades of quality‐adjusted life‐years compared with matched non‐SCD populations [5]. SCD also constitutes a substantial economic burden, including direct costs to the healthcare system and indirect costs associated with patient productivity loss [7, 8, 9, 10]. Costs attributable to management of SCD total more than $1.1 billion annually in the US [11]. In a survey of 187 respondents with SCD, only a third reported being employed and, in these individuals, annual costs due to pain‐related absenteeism and presenteeism have been estimated at $15,103 per person [9].

The primary symptom of SCD is pain, which can be debilitating and tends to become more frequent with age [12]. Patients with SCD often experience chronic pain punctuated by vaso‐occlusive crises, a hallmark complication of SCD that results when vessels become occluded by sickled red blood cells, causing ischemia and inflammation in surrounding tissues [2]. Vaso‐occlusive crises are a primary cause of morbidity among patients with SCD and account for the majority of hospitalizations and emergency department visits in this population [13, 14].

Approaches for reducing the frequency and/or severity of vaso‐occlusive crises and other SCD‐related pain include opioids and other pharmacological therapies, red blood cell transfusion therapy, and disease‐modifying therapies such as hydroxyurea, which is currently the only pharmaceutical treatment for SCD approved for patients as young as 9 months old [15]. In light of substantial evidence that hydroxyurea is safe and efficacious for improving clinical outcomes in SCD [15, 16, 17, 18, 19], the National Heart, Lung, and Blood Institute recommended in 2014 that patients with SCD be offered hydroxyurea treatment regardless of disease severity [15].

While vaso‐occlusive crises are the most frequent manifestation of SCD requiring urgent medical care, SCD‐associated complications are varied and affect a wide range of organ systems [20, 21]. Complications that have been noted by patients as highly relevant but until recently were not as well understood include priapism, nephropathy, and neurologic injury. Although there is some evidence on the incidence of these complications [22, 23, 24], data on the resources and costs associated with their management are sparse and have been limited to Medicaid populations [8]. To address these gaps, we assessed SCD complication rates and associated healthcare resource utilization (HCRU) and costs among 2524 pediatric and adult patients with SCD in a large US administrative claims database including commercially insured as well as Medicare‐enrolled individuals.

## 2 METHODS

### 2.1 Study design and data source

This was a retrospective observational study conducted using administrative claims data from the Optum Research Database (ORD) from July 01, 2013 through March 31, 2020 (study period). The ORD is geographically diverse across the US and contains deidentified medical and pharmacy claims data and linked enrollment information for individuals enrolled in US health plans. Medical claims pertain to both healthcare providers and facilities and include diagnosis and procedure codes from the International Classification of Diseases, 9th and 10th Revisions, Clinical Modification (ICD‐9‐CM and ICD‐10‐CM); Current Procedural Terminology or Healthcare Common Procedure Coding System codes; site of service codes; paid amounts; and other information. Pharmacy claims include drug name, National Drug Code, dosage form, drug strength, fill date, and financial information for outpatient pharmacy services.

### 2.2 Patient selection and cohort assignment

**FIGURE 1:**
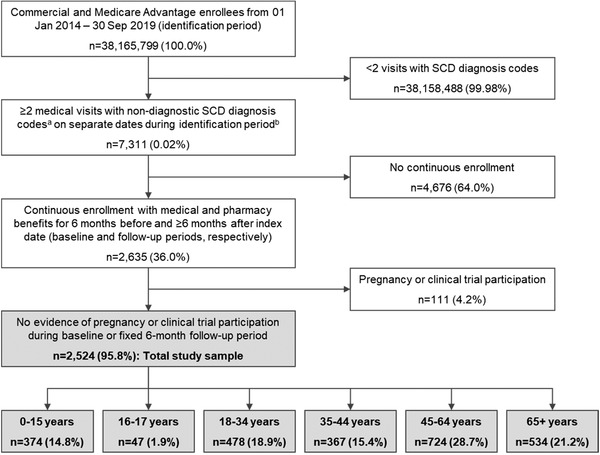
Patient identification and attrition. SCD, sickle cell disease. ^a^SCD diagnosis codes: ICD‐9‐CM 282.41, 282.42, 282.61, 282.62, 282.63, 282.64, 282.68, 282.69, 282.60; ICD‐10‐CM D5740, D57411, D57412, D57419, D5700, D5701, D5702, D5720, D57211, D57212, D57219, D5780, D57811, D57812, D57819, D751. ^b^Patients with missing demographic information were excluded in this step.

The study included patients with ≥2 medical visits with a SCD diagnosis code (see Figure 1 footnote) for nondiagnostic services (i.e., excluding services such as imaging, that are used to diagnose or rule out conditions) on separate dates from January 01, 2014 through September 30, 2019 (identification period). This algorithm has been previously shown to have high positive predictive value for identifying patients with SCD [25, 26]. The index date was defined as the date for the first claim with an SCD diagnosis during the identification period (Figure 1). Patients were required to have continuous enrollment for 6 months before and ≥6 months after the index date (baseline and follow‐up periods, respectively). Patients with evidence of pregnancy or clinical trial participation during the baseline or follow‐up periods were excluded.

### 2.3 Study variables

Baseline demographic and clinical characteristics (age, sex, SCD genotype, Charlson morbidity score [27], and comorbidities identified using Clinical Classifications Software from the Agency for Healthcare Research and Quality [AHRQ] [28]) were assessed during the baseline period.

Study outcomes included SCD treatment patterns (hydroxyurea use, opioid use, red blood cell transfusions); SCD‐related complications (priapism, acute kidney injury, chronic kidney disease, neurologic injury; see Supplemental Table for codes); all‐cause HCRU (ambulatory visits, emergency department visits, inpatient admissions); all‐cause healthcare costs (ambulatory costs, emergency costs, inpatient costs, other medical costs, pharmacy costs), and total healthcare costs related to each SCD complication, which comprised medical claims with diagnosis codes in position 1 or 2 on the claim for SCD‐related complications and pharmacy claims for SCD complication treatments. SCD treatment patterns were assessed during the fixed 6‐month follow‐up period, while complications, HCRU, and healthcare costs were assessed during the variable follow‐up period. HCRU and costs were presented per patient per month (PPPM) to account for variable follow‐up. Costs were calculated as combined patient‐paid and health plan–paid amounts adjusted to 2019 US$ [29].

### 2.4 Statistical analysis

All study variables were analyzed descriptively and stratified by age category.

Differences across study cohorts were evaluated using chi‐square tests for binary measures and analysis of variance for continuous measures. Incidence rates of SCD‐related complications were calculated per 1000 person‐years (PY) with the numerator being the number of patients with new evidence of SCD complications from 2014 through 2019, and the denominator being the number of years at risk for SCD complications from 2014 through 2019 (excluding the 6‐month baseline period). Complication incidence rates were compared among study age cohorts (ages 0–15, 16–17, 18–34, 35–44, 45–64, and 65+) by calculating rate ratios for each age group versus the 0–15 years group.

The cumulative prevalence of patients with each SCD‐related complication (including baseline occurrences) was reported for up to 72 months after the index date, using a Kaplan‐Meier methodology to account for censoring.

Statistical significance was defined as *p* ≤ 0.05. All statistical analyses were performed using SAS 9.4 (SAS Institute, Cary, NC, USA).

## 3 RESULTS

### 3.1 Study sample

Of 7311 patients with SCD diagnosis codes during the identification period, 2524 met the continuous enrollment and exclusion criteria to qualify for study inclusion (Figure 1). Nearly 85% were adults (aged ≥18 years), mean (SD) age was 43.4 (22.4) years, 57.3% were female, and 62.1% had commercial insurance, with the rest enrolled in Medicare (Table 1). The distribution of genotypes was 24.6% Hb‐SS, 8.8% Hb‐SC, and 7.8% Hb‐Sbeta‐thalassemia; the remaining patients had multiple known genotypes (2.5%), other genotypes (3.1%), or unknown genotypes (53.4%). Mean (SD) follow‐up time was 2.7 (1.7) years (Table 1).

**TABLE 1:** Patient characteristics

**Characteristic**	**Total *N* = 2524**	**0–15 years *n* = 374**	**16–17 years *n* = 47**	**18–34 years *n* = 478**	**35–44 years *n* = 367**	**45–64 years *n* = 724**	**65+ years *n* = 534**	***p*‐Value**
Age, years, mean (SD)	43.4 (22.4)	8.7 (4.2)	16.6 (0.5)	26.5 (4.9)	39.5 (2.9)	53.8 (5.7)	73.9 (6.6)	<0.001
Female sex, *n* (%)	1445 (57.3)	188 (50.3)	20 (42.6)	236 (49.4)	211 (57.5)	452 (62.4)	338 (63.3)	<0.001
Insurance type, *n* (%)								
Commercial	1567 (62.1)	374 (100.0)	47 (100.0)	431 (90.2)	283 (77.1)	404 (55.8)	28 (5.2)	<0.001
Medicare	957 (37.9)	0 (0.0)	0 (0.0)	47 (9.8)	84 (22.9)	320 (44.2)	506 (94.8)	<0.001
Geographic region, *n* (%)								
Northeast	305 (12.1)	81 (21.7)	8 (17.0)	77 (16.1)	60 (16.4)	108 (14.9)	94 (17.6)	0.002
South	1668 (66.1)	235 (62.8)	30 (63.8)	312 (65.3)	255 (69.5)	511 (70.6)	325 (60.9)	0.005
Midwest	428 (17.0)	81 (21.7)	8 (17.0)	77 (16.1)	60 (16.4)	108 (14.9)	94 (17.6)	0.130
West	117 (4.6)	17 (4.6)	4 (8.5)	20 (4.2)	12 (3.3)	37 (5.1)	27 (5.1)	0.556
Other	6 (0.2)	3 (0.8)	0 (0.0)	1 (0.2)	2 (0.5)	0 (0.0)	0 (0.0)	0.087
Genotype^a^, *n* (%)								
Hb‐SS	620 (24.6)	132 (35.3)	21 (44.7)	172 (36)	89 (24.3)	140 (19.3)	66 (12.4)	<0.001
Hb‐SC	221 (8.8)	67 (17.9)	4 (8.5)	40 (8.4)	27 (7.4)	48 (6.6)	35 (6.6)	<0.001
Hb‐SThalassemia	196 (7.8)	34 (9.1)	4 (8.5)	27 (5.7)	29 (7.9)	47 (6.5)	55 (10.3)	0.065
Multiple known types	62 (2.5)	17 (4.6)	2 (4.3)	18 (3.8)	10 (2.7)	10 (1.4)	5 (0.9)	0.001
Other	77 (3.1)	13 (3.5)	3 (6.4)	17 (3.6)	5 (1.4)	17 (2.4)	22 (4.1)	0.101
Unspecified	1348 (53.4)	111 (29.7)	13 (27.7)	204 (42.7)	207 (56.4)	462 (63.8)	351 (65.7)	<0.001
Charlson comorbidity score category, n (%)								
0	1574 (62.4)	318 (85)	41 (87.2)	400 (83.7)	263 (71.7)	375 (51.8)	177 (33.2)	<0.001
1–2	592 (23.5)	53 (14.2)	5 (10.6)	64 (13.4)	83 (22.6)	216 (29.8)	171 (32.0)	<0.001
3–4	220 (8.7)	1 (0.3)	1 (2.1)	9 (1.9)	14 (3.8)	87 (12.0)	108 (20.2)	<0.001
5+	138 (5.5)	2 (0.5)	0 (0.0)	5 (1.1)	7 (1.9)	46 (6.4)	78 (14.6)	<0.001
Top AHRQ comorbidities, *n* (%)^b^								
Anemia	1294 (51.3)	197 (52.7)	22 (46.8)	217 (45.4)	196 (53.4)	372 (51.4)	290 (54.3)	0.078
Hypertension	929 (36.8)	5 (1.3)	0 (0.0)	37 (7.7)	89 (24.3)	350 (48.3)	448 (83.9)	<0.001
Diseases of the heart	879 (34.8)	33 (8.8)	8 (17.0)	101 (21.1)	113 (30.8)	308 (42.5)	316 (59.2)	<0.001
Other lower respiratory diseases^c^	741 (29.4)	73 (19.5)	6 (12.8)	95 (19.9)	98 (26.7)	243 (33.6)	226 (42.3)	<0.001
Other connective tissue diseases^d^	732 (29.0)	24 (6.4)	6 (12.8)	97 (20.3)	94 (25.6)	270 (37.3)	241 (45.1)	<0.001
Diseases of the urinary system^e^	710 (28.1)	30 (8.0)	5 (10.6)	71 (14.9)	78 (21.3)	243 (33.6)	283 (53.0)	<0.001
Follow‐up time, years, mean (SD)^f^	2.7 (1.7)	3.1 (1.9)	3.2 (2.1)	2.5 (1.7)	2.8 (1.8)	2.7 (1.7)	2.5 (1.4)	<0.001

Abbreviations: AHRQ, Agency for Healthcare Research and Quality; SD, standard deviation.

^a^ Genotype is mutually exclusive; patients with a known genotype may have had other or unspecified types.

^b^ Identified using Clinical Classifications Software from the Agency for Healthcare Research and Quality.[28]

^c^ Lower respiratory diseases other than chronic obstructive pulmonary disease, asthma, aspiration pneumonitis, pleurisy, respiratory failure, or lung disease due to external agents.

^d^ Connective tissue diseases other than systemic lupus erythematosus.

^e^ Nephritis, chronic kidney disease, calculus of bladder/kidney, nephrotic syndrome, among other conditions.

^f^ Adjusted for death.

Anemia was the most prevalent baseline AHRQ comorbidity overall (51.3% of patients) and was common across age groups (Table 1). Among pediatric patients, other common comorbidities included lower respiratory diseases (19.5% for 0–15 years, 12.8% for 16–17 years) and diseases of the heart (8.8% for 0–15 years, 17.0% for 16–17 years). Among the oldest patients (65+ years), commonly reported comorbidities included hypertension (83.9%), diseases of the heart (59.2%), diseases of the urinary system (53.0%), connective tissue diseases (45.1%), and lower respiratory diseases (42.3%). The prevalence of comorbidities except for anemia differed by age (*p* < 0.001).

### 3.2 SCD treatment patterns

SCD medication use during follow‐up varied significantly across age groups (*p* < 0.001), with the most common medications being short‐acting opioids (54.0%) followed by hydroxyurea (11.0%) and long‐acting opioids (6.9%) (Table 2). Use of hydroxyurea (11.0% overall) was highest among patients aged 16–17 years (25.5%) and very low among the oldest patients (1.1%). Short‐acting opioid use increased with age (from 35.6% of patients aged 0–15 years to 62.7% of patients aged 45–64 years), with the exception of the 65+ age group (45.1%). Use of long‐acting opioids was low overall (6.9%), highest among patients aged 35–44 years (11.4%), and lowest among the youngest and oldest patients (1.6% and 2.3%, respectively).

**TABLE 2:** Follow‐up sickle cell disease treatments

Treatment^a^, *n* (%)	Total *N* = 2524	**0–15 years *n* = 374**	**16–17 years *n* = 47**	**18–34 years *n* = 478**	**35–44 years *n* = 367**	**45–64 years *n* = 724**	**65+ years *n* = 534**	***p*‐Value**
Hydroxyurea	278 (11.0)	67 (17.9)	12 (25.5)	78 (16.3)	47 (12.8)	68 (9.4)	6 (1.1)	<0.001
Opioids (short‐acting)	1362 (54.0)	133 (35.6)	17 (36.2)	290 (60.7)	227 (61.9)	454 (62.7)	241 (45.1)	<0.001
Opioids (long‐acting)	175 (6.9)	6 (1.6)	2 (4.3)	41 (8.6)	42 (11.4)	72 (9.9)	12 (2.3)	<0.001
Red blood cell transfusion	400 (15.9)	56 (15.0)	10 (21.3)	84 (17.6)	62 (16.9)	118 (16.3)	70 (13.1)	0.333

^a^ Crizanlizumab, L‐glutamine, and voxelotor were approved near the end of the data extraction period for this analysis and were each used by only 0–1 patients during follow‐up.

Red blood cell transfusion was observed among 15.9% of patients overall and did not vary by age (*p* = 0.333) (Table 2).

### 3.3 Incidence and prevalence of SCD‐related complications

In the overall population, incidence per 1000 PY during follow‐up was highest for acute kidney injury (53.1), followed by chronic kidney disease (40.6) and stroke (39.0) (Table 3). Acute kidney injury and chronic kidney disease were the only complications reported among patients aged 16–17 years (20.2 per 1000 PY and 13.5 per 1000 PY, respectively). The incidence rate of acute kidney injury increased dramatically with age; compared with patients aged 0–15 years, rate ratios for this complication ranged from 11.5 (*p* = 0.014) for patients aged 16–17 to 69.7 (*p* < 0.001) for those aged 65+ (Table 4).

**TABLE 3:** Follow‐up incidence rates of sickle cell disease complications

	**Total *N* = 2524**		**0–15 years *n* = 374**		**16–17 years *n* = 47**		**18–34 years *n* = 478**		**35–44 years *n* = 367**		**45–64 years *n* = 724**		**65+ years *n* = 534**	
**Complication**	**Number at risk**	**Rate per 1000 PY**	**Number at risk**	**Rate per 1000 PY**	**Number at risk**	**Rate per 1,000 PY**	**Number at risk**	**Rate per 1000 PY**	**Number at risk**	**Rate per 1000 PY**	**Number at risk**	**Rate per 1000 PY**	**Number at risk**	**Rate per 1000 PY**
Priapism^a^	1072	7.9	185	7.6	27	0.0	238	19.9	154	14.5	272	1.4	196	0.0
Acute kidney injury	2404	53.1	372	1.8	47	20.2	469	32.2	355	37.2	685	71.9	476	122.4
CKD	2306	40.6	373	8.0	47	13.5	472	10.5	358	23.2	638	45.5	418	136.0
Neurologic injury	2395	41.7	369	17.7	46	0.0	470	27.6	353	24.2	688	49.7	469	91.8
Stroke	2406	39.0	369	15.7	46	0.0	470	23.8	354	23.0	692	47.9	475	85.7
TIA	2493	16.7	374	6.2	47	0.0	477	7.8	363	10.1	712	19.9	520	37.9
Neurocog. deficit	2520	3.4	374	0.9	47	0.0	478	1.7	367	2.0	724	5.1	530	6.2

Abbreviations: CKD, chronic kidney disease; neurocog., neurocognitive; PY, person‐years; TIA, transient ischemic attack.

^a^ Among patients identified as male.

**TABLE 4:** Follow‐up incidence rate ratios of sickle cell disease complications

	**16–17 years *n* = 47**		**18–34 years *n* = 478**		**35–44 years *n* = 367**		**45–64 years *n* = 724**		**65+ years *n* = 534**	
**Complication**	**Rate ratio versus ≤15 years**	***p*‐Value**	**Rate ratio versus ≤15 years**	***p*‐Value**	**Rate ratio versus ≤15 years**	***p*‐Value**	**Rate ratio versus ≤15 years**	***p*‐Value**	**Rate ratio versus≤15 years**	***p*‐Value**
Priapism^a^	0.0	0.549	2.6	0.094	1.9	0.333	0.1824	0.118	0.0	0.078
Acute kidney injury	11.5	0.014	18.3	<0.001	21.2	<0.001	40.9250	<0.001	69.7	<0.001
CKD	1.7	0.499	1.3	0.540	2.9	0.005	5.6988	<0.001	17.0	<0.001
Neurologic injury	0.0	0.089	1.6	0.129	1.4	0.313	2.8084	<0.001	5.2	<0.001
Stroke	0.0	0.116	1.5	0.188	1.5	0.246	3.0442	<0.001	5.4	<0.001
TIA	0.0	0.413	1.3	0.660	1.6	0.329	3.1973	0.002	6.1	<0.001
Neurocog. deficit	0.0	0.883	2.0	0.635	2.3	0.553	5.8780	0.052	7.1	0.032

Abbreviations: CKD, chronic kidney disease; neurocog., neurocognitive; TIA, transient ischemic attack.

^a^ Among patients identified as male.

Chronic kidney disease was higher only among patients aged 35 years and older compared with those aged 0–15 years (*p* < 0.05 for all), while neurologic injury was higher only among patients aged 45–64 years and 65+ years compared with 0–15 years (*p* < 0.001 for both).

**FIGURE 2:**
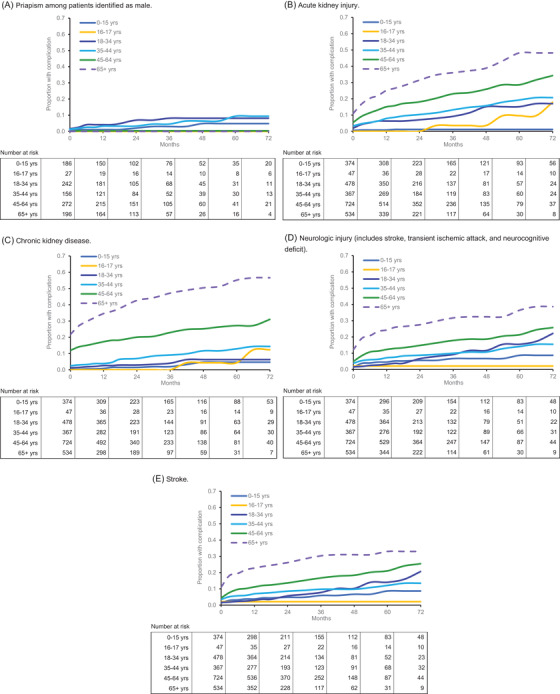
Kaplan‐Meier analysis of cumulative prevalence of sickle cell disease complications. For all panels, log‐rank *p* < 0.001. (A) Priapism among patients identified as male. (B) Acute kidney injury. (C) Chronic kidney disease. (D) Neurologic injury (includes stroke, transient ischemic attack, and neurocognitive deficit). (E) Stroke.

The cumulative prevalence of patients with each SCD‐related complication during follow‐up, including baseline occurrences and accounting for censoring, is shown in Figure 2. The prevalence of all conditions differed significantly by age (*p* < 0.001 for all). With the exception of priapism, the prevalence of comorbidities was highest for patients aged 65+ throughout follow‐up, followed by patients aged 45–64.

### 3.4 All‐cause healthcare resource utilization and costs

**FIGURE 3:**
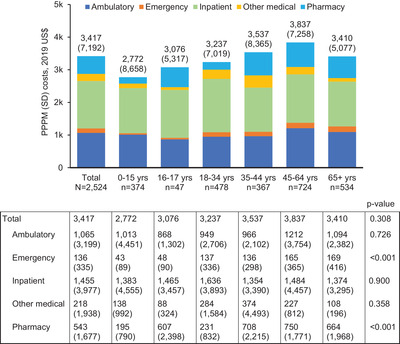
Follow‐up all‐cause per‐patient‐per‐month healthcare costs. Medical costs comprise ambulatory, emergency, inpatient, and other medical costs (costs for services not typically part of an office visit, such as laboratory services). Standard deviations are given in parentheses. PPPM, per‐patient‐per‐month; SD, standard deviation.

Follow‐up PPPM HCRU—including ambulatory visits, emergency department visits, inpatient stays, inpatient days, and pharmacy fills—all differed significantly across age groups, increasing with age (*p* < 0.001 for all) (Table 5). Outpatient utilization was particularly high among the oldest study patients (65+ years), with a mean (SD) of 4.0 (4.1) ambulatory visits per patient each month. Total follow‐up PPPM (SD) healthcare costs were $3417 ($7192) for the overall population, attributable primarily to medical costs and to inpatient costs in particular ($1455) (Figure 3). Only emergency costs and pharmacy costs differed significantly by age (*p* < 0.001 for both) (Figure 3). Total follow‐up PPPM (SD) healthcare costs among patients with each type of SCD complication were $893 ($2936) for priapism, $1612 ($5125) for acute kidney injury, $2404 ($7064) for chronic kidney disease, and $1338 ($4088) for any neurologic injury (which comprised $1390 [$4176] for stroke, $333 [$842] for transient ischemic attack, and $915 [$2361] for neurocognitive deficit).

**TABLE 5:** Follow‐up all‐cause per‐patient‐per‐month healthcare resource utilization

**PPPM count, mean (SD)**	**Total *N* = 2524**	**0–15 years *n* = 374**	**16–17 years *n* = 47**	**18–34 years *n* = 478**	**35–44 years *n* = 367**	**45–64 years *n* = 724**	**65+ years *n* = 534**	***p*‐Value**
Ambulatory visits	2.5 (3.1)	1.5 (1.7)	1.5 (1.5)	1.6 (1.8)	2.0 (2.5)	2.9 (3.3)	4.0 (4.1)	<0.001
Emergency department visits	0.3 (0.6)	0.1 (0.2)	0.1 (0.2)	0.3 (0.5)	0.3 (0.5)	0.3 (0.5)	0.3 (0.9)	<0.001
Inpatient stays	0.1 (0.1)	0.1 (0.1)	0.1 (0.1)	0.1 (0.2)	0.1 (0.1)	0.1 (0.1)	0.1 (0.1)	<0.001
Inpatient days among patients with ≥1 inpatient stay	1.2 (2.2)	0.5 (0.8)	0.8 (1.1)	1.3 (2.5)	1.1 (1.9)	1.2 (2.1)	1.6 (2.5)	<0.001
Pharmacy fills	2.6 (3.1)	1.0 (1.2)	1.0 (1.5)	1.3 (1.6)	2.3 (2.8)	3.5 (3.6)	3.9 (3.5)	<0.001

Abbreviations: PPPM, per‐patient‐per‐month; SD, standard deviation.

## 4 DISCUSSION

In this retrospective US claims analysis of insured pediatric and adult patients with SCD, complications were prevalent and occurred across all age groups, including among the youngest patients. Treatments that are typically prescribed to alleviate pain and treat SCD complications—including opioid use and blood transfusions—were also observed, demonstrating substantial disease burden. All‐cause HCRU and costs were similarly high for all age groups and were driven primarily by inpatient stays, which has been observed in previous studies of patients with SCD [10, 11, 30].

Our findings are congruent with existing data demonstrating substantial morbidity due to complications among individuals of all ages with SCD [31, 32, 33, 34]. The prevalence of SCD complications tends to increase over time and is therefore higher among older patients [23, 24]—a phenomenon also observed in our analysis, with the exception of priapism. However, earlier studies have also shown that even younger patients with SCD already carry a substantial burden of SCD‐related complications, including cerebrovascular disease, pulmonary disease, hepatic disease, nephropathy, and neurological disorders [31, 32, 33, 34, 35]. In one retrospective claims analysis of 1186 adolescents with SCD, 61.1% of the study population was found to have at least one chronic SCD‐related complication during a 1‐year follow‐up [31]. Accordingly, cognitive deficits among children with SCD have been observed beginning at preschool age and persisting throughout life [33, 35], and pathophysiological changes associated with sickle cell nephropathy have been identified as early as infancy [34]. The low rate of cognitive defects reported among younger patients in our study could potentially reflect the lack of a specific ICD code for silent cerebral infarcts and nonadherence to guideline‐based systematic screening for neurocognitive defects [12]. The substantial prevalence of complications in younger age groups suggests that individuals with SCD will face a high burden of morbidity and associated costs over their lifespan. Indeed, an analysis of 4294 pediatric and adult Medicaid enrollees with SCD indicated that the lifetime cost of care would average $460,151 per patient in 2005 US dollars [11].

Importantly, the accumulation of damage from repeated episodes of vaso‐occlusion as patients age [36] likely lays the groundwork for the extremely high complication rates observed among older patients in our analysis and others [23, 24]. Observational studies suggest that progression of complications is inevitable for most patients with SCD, with nearly half of this population exhibiting irreversible organ damage due to chronic vasculopathy by the 5th decade of life [24]. More recent prospective data from the US are lacking; however, among a prospectively followed cohort of adult patients with SCD in the Netherlands, 80% had at least one form of SCD‐related organ damage after 7 years of follow‐up, and 62% had developed a new form of organ damage during the same period [23]. Taken together, these findings highlight the consequences of a lifetime of chronic vaso‐occlusion and indicate that more aggressive treatment management among younger patients may be warranted to reduce their current and future morbidity burden.

The mainstays of treatment for preventing vaso‐occlusive crises among patients with SCD have been red blood cell transfusions and hydroxyurea [15], each of which was used by a relatively small percentage of patients in our study—only 15.9% and 11.0% of the overall patient population, respectively. Red blood cell transfusion, while effective for reducing morbidity in SCD, is also associated with a variety of adverse reactions, some of which can be severe [37, 38]. Consequently, guidelines for SCD management stress the need for risk‐benefit analysis when deciding whether to use transfusion therapy and explicitly recommend against it in certain settings, including uncomplicated vaso‐occlusive crises, priapism, asymptomatic anemia, and acute kidney injury in the absence of multisystem organ failure [15]. In contrast, current guidelines suggest that hydroxyurea should be offered to nearly all patients with SCD [15] on the strength of considerable evidence supporting its efficacy, tolerability, and favorable safety profile [16, 17, 18, 19]. Notably, however, we found that hydroxyurea use among younger patients, while higher than that in older age groups, remained strikingly low. Only 17.9% of patients aged 0–15 and 25.5% of those aged 16–17 had a fill for hydroxyurea during follow‐up, comparable to the approximately 20%–33% observed in other retrospective analyses of children and adults with SCD [39, 40, 41].

In view of the abundant evidence that hydroxyurea treatment is associated with not only improved clinical and economic outcomes [42, 43, 44] but also higher health‐related quality of life among patients with SCD [45, 46, 47, 48], the potential underuse of hydroxyurea observed in the present study may reflect lost opportunity for slowing the progression of SCD complications in this patient population—particularly considering that opioid use was high, implying a substantial disease burden [49]. The development and utilization of novel treatments targeting the underlying pathologic processes of vaso‐occlusion is an important facet of addressing this gap [50], but our results suggest that examination of approaches that could mitigate barriers to hydroxyurea use and adherence is also warranted. Pediatric and adult studies have identified multiple barriers that contribute to low hydroxyurea utilization among patients with SCD, including patient forgetfulness, difficulty obtaining refills, lack of access to quality healthcare and/or specialist care, and concerns about effectiveness and side effects on the part of patients and providers alike [51]. While high‐quality studies evaluating interventions designed to increase utilization of hydroxyurea have thus far been lacking [52, 53], several relevant trials are currently underway [54, 55].

### 4.1 Study limitations

This study has several limitations. First, self‐reported race/ethnicity data are not available in the ORD and could not be presented for this analysis. Second, the presence of a claim for a filled prescription does not indicate that the medication was taken as prescribed; and medications filled over‐the‐counter, provided as samples by a physician, or received through patient support programs are not observed in claims data. In addition, the prevalence of neurocognitive deficit may have been underestimated, as this complication is not fully captured by claims data. Third, hydroxyurea use was not captured by SCD genotype, and many study patients had unknown genotypes; therefore, the degree to which the potential HU underuse observed in this study occurred among patients with severe disease is not known. Fourth, analysis of treatment patterns does not include newer medications such as crizanlizumab, L‐glutamine, and voxelotor, which were approved near the end of the data extraction period and each used by only 0‐1 patients during follow‐up. Finally, because all study patients were enrolled in a commercial or Medicare Advantage health plan during the study period, findings may not be generalizable to patients who are uninsured or enrolled in other health plans.

## 5 CONCLUSION

With the use of current treatments, patients with SCD across the age spectrum had a high burden of complications, associated with substantial HCRU and costs; however, therapies with the potential to reduce disease progression were underused. Our findings suggest unmet needs for treatment management among patients with SCD.

## CONFLICT OF INTEREST

Deepa Manwani has served as a consultant for Novartis. Arthur Burnett has served as a consultant for Novartis; received grants from Boston Scientific, Futura Medical, Myriad Genetics, Comphya SA, National Institutes of Health, and Endo Pharmaceuticals; has participated in the PhenX Sickle Cell Disease Genitourinary Working Group; and has provided leadership to the Urology Care Foundation and Mentoring Male Teens in the Hood. Sara Wang is an employee of Optum, which was contracted by Novartis to conduct this study. Tanya Burton was an employee of Optum at the time this study was conducted. Amy Anderson owns stock in UnitedHealth Group and is an employee of Optum, which was contracted by Novartis to conduct the study and is a subsidiary of UnitedHealth Group. Jincy Paulose, Glorian Yen, and Soyon Lee are employees of and own stock in Novartis. Santosh Saraf has served as a consultant for Novartis, Global Blood Therapeutics, FORMA, and Agios and has served on a speakers bureau for Global Blood Therapeutics.

## ETHICS STATEMENT

This study was conducted in accordance with the principles of the Declaration of Helsinki. Because no identifiable protected health information was accessed, institutional review board approval or waiver of approval was not required.

## Supporting information

Supplemental Table. Disease codes

## Data Availability

Research data are not shared because the Optum Research Database contains propriety elements owned by Optum.
