# Supplementary material for: Treatment patterns and burden of complications associated with sickle cell disease: A US retrospective claims analysis
Source: EJHaem. 2022 Oct 6;3(4):1135–44. doi: 10.1002/jha2.575 (PMC9713207; doi:10.1002/jha2.575)
Supplement: Supplementary file 1 — Supplemental Table. Disease codes [file JHA2-3-1135-s001.docx]

**Supplemental Table.** Disease Codes

| Condition | Code type | Code(s) |
| --- | --- | --- |
| Acute kidney injury | ICD-9-CM Dx | 580.0, 580.4, 580.81, 580.89, 580.9, 581.0, 581.1, 581.2, 581.3, 581.81, 581.89, 581.9, 583.0, 583.1, 583.2, 583.4, 583.6, 583.7, 583.81, 583.89, 583.9, 584.5, 584.6, 584.7, 584.8, 584.9 |
|  | ICD-10-CM Dx | N000, N001, N002, N003, N004, N005, N006, N007, N008, N009, N010, N011, N012, N013, N014, N015, N016, N017, N018, N019, N040, N041, N042, N043, N044, N045, N046, N047, N048, N049, N050, N051, N052, N053, N054, N055, N056, N057, N058, N059, N062, N063, N064, N065, N067, N12, N170, N171, N172, N178, N179 |
| Chronic kidney disease | ICD-9-CM Dx | 403.01, 403.11, 403.91, 404.02, 404.03, 404.12, 404.13, 404.92, 404.93, 585.5, 585.6, 585.1, 585.2, 403.00, 403.10, 403.90, 404.00, 404.01, 404.10, 404.11, 404.90, 404.91, 585.3, 585.4 |
|  | ICD-10-CM Dx | I120, I1311, I132, N185, N186, N19, N990, N181, N182, I129, I130, I1310, N183, N184 |
| Priapism | ICD-9-CM Dx | 607.3 |
|  | ICD-10-CM Dx | N4830, N4831, N4832, N4833, N4839 |
| Stroke and neurocognitive deficit | ICD-9-CM Dx | 436, 430, 431, 432.9, 433.01, 433.11, 433.21, 433.31, 433.81, 433.91, 434.01, 434.11, 434.91 |
|  | ICD-10-CM Dx | G463, G464, G465, G466, G467, I638, I639, I6781, I6930, I6931, I69310, I69311, I69312, I69313, I69314, I69315, I69318, I69319, I69320, I69321, I69322, I69323, I69328, I69331, I69332, I69333, I69334, I69339, I69341, I69342, I69343, I69344, I69349, I69351, I69352, I69353, I69354, I69359, I69361, I69362, I69363, I69364, I69365, I69369, I69390, I69391, I69392, I69393, I69398, I6000, I6001, I6002, I6010, I6011, I6012, I602, I6020, I6021, I6022, I6030, I6031, I6032, I604, I6050, I6051, I6052, I606, I607, I608, I609, I610, I611, I612, I613, I614, I615, I616, I618, I619, I6200, I6201, I6202, I6203, I621, I629, I6900, I6901, I69010, I69011, I69012, I69013, I69014, I69015, I69018, I69019, I69020, I69021, I69022, I69023, I69028, I69031, I69032, I69033, I69034, I69039, I69041, I69042, I69043, I69044, I69049, I69051, I69052, I69053, I69054, I69059, I69061, I69062, I69063, I69064, I69065, I69069, I69090, I69091, I69092, I69093, I69098, I6910, I6911, I69110, I69111, I69112, I69113, I69114, I69115, I69118, I69119, I69120, I69121, I69122, I69123, I69128, I69131, I69132, I69133, I69134, I69139, I69141, I69142, I69143, I69144, I69149, I69151, I69152, I69153, I69154, I69159, I69161, I69162, I69163, I69164, I69165, I69169, I69190, I69191, I69192, I69193, I69198, I6920, I6921, I69210, I69211, I69212, I69213, I69214, I69215, I69218, I69219, I69220, I69221, I69222, I69223, I69228, I69231, I69232, I69233, I69234, I69239, I69241, I69242, I69243, I69244, I69249, I69251, I69252, I69253, I69254, I69259, I69261, I69262, I69263, I69264, I69265, I69269, I69290, I69291, I69292, I69293, I69298, I6300, I63011, I63012, I63013, I63019, I6302, I63031, I63032, I63033, I63039, I6309, I6310, I63111, I63112, I63113, I63119, I6312, I63131, I63132, I63133, I63139, I6319, I6320, I63211, I63212, I63213, I63219, I6322, I63231, I63232, I63233, I63239, I6329, I6330, I63311, I63312, I63313, I63319, I63321, I63322, I63323, I63329, I63331, I63332, I63333, I63339, I63341, I63342, I63343, I63349, I6339, I6340, I63411, I63412, I63413, I63419, I63421, I63422, I63423, I63429, I63431, I63432, I63433, I63439, I63441, I63442, I63443, I63449, I6349, I6350, I63511, I63512, I63513, I63519, I63521, I63522, I63523, I63529, I63531, I63532, I63533, I63539, I63541, I63542, I63543, I63549, I6359, I636 |
| Transient ischemic attack | ICD-9-CM Dx | 362.34, 435.0, 435.1, 435.2, 435.3, 435.8, 435.9 |
|  | ICD-10-CM Dx | G450, G451, G452, G453, G454, G458, G459, G460, G461, G462, G468 |

Dx, diagnosis; ICD-9-CM, International Classification of Diseases, 9th Revision, Clinical Modification; ICD-10-CM, International Classification of Diseases, 10th Revision, Clinical Modification.
